# 血液生态：从概念到实践

**DOI:** 10.3760/cma.j.cn121090-20251229-00621

**Published:** 2026-01

**Authors:** 涛 程, 洪 王

**Affiliations:** 1 中国医学科学院血液病医院（中国医学科学院血液学研究所），血液与健康全国重点实验室，国家血液系统疾病临床医学研究中心，天津 300020 State Key Laboratory of Experimental Hematology, National Clinical Research Center for Blood Diseases, Institute of Hematology & Blood Diseases Hospital, Chinese Academy of Medical Sciences & Peking Union Medical College, Tianjin 300020, China; 2 天津医学健康研究院，天津 301600 Tianjin Institutes of Health Science, Tianjin 301600, China

## Abstract

血液生态概念的提出，标志着血液学研究正从传统的细胞或器官分析模式，转向以整体性、关联性、开放性和动态性为特征的跨器官互作研究范式。该框架强调血液作为整合全身稳态的动态耦合网络地位，利用单细胞多组学、空间组学及人工智能等技术，实现了对血液生态景观前所未有的深度解析与系统性测量。血液生态研究不仅推动了血液病的精准诊疗，更拓展至非血液系统疾病领域，通过识别循环分子、克隆性造血等机制，揭示了跨器官的病理演化规律，为未来实现“系统预测—智能干预—生态重建”的精准医学闭环奠定了基础。

## Abstract (en)

The introduction of the blood ecosystem concept marks a paradigm shift in hematology from traditional cell- or organ-centered analyses toward a framework focused on cross-organ interactions characterized by systemic integration, connectivity, openness, and dynamic regulation. This framework emphasizes blood as a dynamically coupled network that integrates whole-body homeostasis, rather than a collection of isolated components. Enabled by advances in single-cell multi-omics, spatial profiling, and artificial intelligence-based analytical approaches, the blood ecosystem can now be interrogated with unprecedented resolution and quantified at the system level. Blood ecosystem research has not only advanced precision diagnosis and treatment of hematologic diseases, but has also expanded into non-hematological disorders by elucidating mechanisms such as circulating molecular signaling and clonal hematopoiesis, thereby uncovering principles of cross-organ pathological evolution. Together, this framework provides a foundation for the development of a precision medicine paradigm centered on system-level prediction, intelligent intervention, and ecological reconstruction.

一、传统血液系统框架的局限性与范式更新的必然性

自经典血液学建立以来，“血液系统”被界定为由血液及造血器官构成的生理系统，其内涵主要基于器官结构和细胞来源框架，强调造血部位、细胞谱系、成熟路径及循环通路。这一定义奠定了血液学百年来的发展基础。然而，其理论范式建立在将血液成分视作相对独立的功能单位前提之上，假定细胞行为主要由细胞内源决定；血液成分之间的动态互作、血液循环微环境、血流机械应力以及血浆因子等外源调控因素并未系统纳入。血液被理解为运输介质，而非作为整合全身稳态的系统性器官；血液作为贯穿全身的流动中枢，其介导的跨器官能量、物质与信息交换网络及其对机体稳态的系统性调控，长期以来被严重低估。

过去十年，单细胞多组学、空间组学、多维流式与循环免疫组学、cfDNA/RNA与细胞外囊泡（EV）组学、大规模蛋白质组学和代谢组学等技术突飞猛进，使我们能够以前所未有的深度解析血液中细胞与非细胞成分的多尺度互作网络，并揭示血液在跨器官通讯、稳态维持与病理转化中的核心地位。

基于上述认识，我们在五年前首次提出“血液生态（Blood Ecosystem）”的概念[1]，并在随后的研究中不断加以拓展与深化[2]。血液生态定义为：由血液系统中的细胞与非细胞成分及其与全身组织器官之间通过物质、能量和信息流动所构成的开放性动态互作网络，该网络在机体稳态维持及病理转化过程中发挥整体性调控作用。这一定义强调血液并非由相互独立成分构成的静态集合，而是一个具有系统性、动态性与高度互作特征的功能整体，其结构与功能依赖于多层级要素之间持续而可塑的相互作用。

血液生态研究旨在系统解析三个层面的核心问题：其一，血液系统内部由细胞、蛋白质、核酸、代谢物及EV等构成的多层级互作网络及其调控机制；其二，血液生态网络与全身器官之间的双向调控关系，包括免疫、代谢、凝血与信号转导等跨系统通讯过程；其三，血液生态稳态的建立、维持与破坏机制，阐明在疾病、衰老及环境应激条件下生态网络的重塑、解耦或崩解及其系统性后果。

血液生态的提出不仅拓展了传统血液系统的内涵，也为理解机体稳态调控和疾病发生机制提供了新的理论框架，推动血液学研究从以细胞或器官为中心的分析模式，迈向以系统、网络和跨器官互作为核心的研究范式。

二、从概念到实践，血液生态研究近五年的关键进展

过去五年，随着技术范式从群体分析迈向单细胞与多组学整合，血液生态研究迅速推进。研究重点从描述笼统的平均细胞状态转变为精细解析单细胞异质性及细胞-分子互作网络。构建人血细胞图谱（Atlas of Blood Cells, ABC），为稳态生态描绘了基线框架[3]，并通过ABC Portal实现多组学数据标准化与细胞间通讯分析，使跨研究整合成为可能[4]。单细胞组学进一步揭示发育、稳态与应激状态下的动态生态特征：胚胎期巨核细胞存在多亚型，成体造血中可精确识别单能中性粒细胞前体；共同反映造血补充机制的高度精细调控[5]–[7]。在急性应激如脓毒症或感染中，中性粒细胞祖细胞快速扩增以维持免疫平衡，说明血液生态具备快速响应与适应能力。

多组学临床动态监测进一步展示了血液生态在感染压力下的重塑，例如对病毒突破感染大队列样本的纵向分析，揭示了血小板的抗病毒“非经典”作用和免疫记忆衰减轨迹[8]。造血干细胞移植（Hematopoietic stem cell transplantation, HSCT）则为观察生态重建提供了窗口：早期造血优先恢复粒系，且高表达S100A的免疫调节性中性粒细胞前体数量可预测急性移植物抗宿主病（aGVHD）风险[9]。血液生态研究还揭示了红系细胞等非传统免疫成员的免疫调节功能[10]，这一发现进一步拓展了我们对血液生态网络中成员互作复杂性的认知边界。

血液生态正成为理解全身性疾病与实现精准诊疗的重要理论基础。结合单细胞与多组学数据、动态互作解析和机器学习模型，已在疾病风险评估和临床决策支持中初显成效[11]–[12]。然而，血液生态仍仅被部分揭示：其动态平衡机制、跨疾病泛化规律及标准化多模态大数据仍待构建。未来需扩大样本规模与疾病类型，提升纵向与多尺度数据质量，推动血液生态从描述走向可预测、可干预，实现精准临床转化。

三、从ABC计划到HEMO ABC工程，绘制生态基准，解码疾病演化路径

为推动我国血液学跨越式发展并加速精准医学转化，中国医学科学院血液病医院联合多家机构启动ABC计划[1]，系统绘制了从胚胎发育、稳态到感染、炎症和移植等应激状态下的人血液细胞动态图谱，揭示了生态成员间复杂互作，为理解全身性疾病提供系统性基础[5]–[10],[13]。

目前该计划进入第二期——HEMO ABC工程，通过十亿级规模细胞多组学解析，从基因组、表观组、转录组、蛋白组与时空组学等多维度构建动态血液生态图谱，不再停留于静态描述，而致力于构建可计算、可模拟的“数字孪生血液生态”。

HEMO ABC工程的核心使命在于构建开放共享的多组学数据库以及开发基于机器学习的生态预测模型，为全球研究者提供数据基础设施与智能分析能力。该工程有望成为未来精准医学的引擎，为攻克血液病及系统性疾病奠定战略支撑。

四、多模态计算与数字孪生血液生态，人工智能赋能血液生态研究

随着高质量多组学和多维血液数据的积累，AI将深度赋能血液生态研究并显著提升解析效率。血液生态的复杂性在于数据维度高、动态变化快，涉及单细胞、蛋白质组、代谢组和表观遗传等信息，人脑难以整合。AI尤其是深度学习与机器学习模型，擅长在高维、多模态数据中识别隐含规律，为血液生态研究提供关键手段。

当拥有足够规模的血液多组学数据（例如扩展版ABC portal整合临床、影像及各组学数据）时，AI可构建高度仿真的“数字孪生血液生态”，从单细胞模拟迈向系统级动态仿真，显著减少湿实验成本，加速研究迭代。AI还能自动识别细胞互作模式，如在病毒突破感染中发现血小板与白细胞互作网络，捕捉传统统计分析难以识别的复杂模式。

AI还展示出实现精准预警的潜力。例如针对原发性免疫性血小板减少症（ITP）患者中罕见但致命的重症出血风险，AI模型表现出了巨大潜力，为ITP的风险管理提供了标准化的数字治理方案[14]。有前瞻性临床研究使用自主AI智能体daGOAT预测严重aGVHD发生概率，并开具预防处方，证明AI可将复杂血液生态数据转化为可执行的临床决策[15]。

未来，AI有望成为血液生态临床智能助手，通过模拟不同治疗方案（化疗、免疫疗法剂量等）对血液生态网络的影响，为个体提供最优治疗策略。血液生态大数据是燃料，AI是驱动引擎。随着数据规模扩大、模型优化，研究甚至临床管理将从经验驱动转向系统预测模式，实现精准掌控血液疾病乃至全身性疾病的发生发展。

五、血液生态驱动精准诊疗升级：迈向诊断与治疗新范式

随着血液生态概念的不断具象化和可测量化，疾病诊疗正从传统着重细胞或分子“组件层面”的局部干预，转向基于血液生态“系统整体”状态的精准医学。血液生态研究揭示，血液内细胞、非细胞组分与循环微环境之间存在复杂而动态的互作网络，这一网络在稳态维持、免疫调控、能源分配以及病理演化过程中发挥整合作用。由此，精准诊疗不再局限于靶向单一细胞或信号通路，而需关注血液生态整体状态变化，并基于系统层面的失衡特征制定干预策略。

在诊断方面，血液生态研究正在推动血液疾病从依赖传统单一指标向系统状态识别转变。多组学和单细胞技术使解析多维互作网络成为可能，可在临床症状明显出现前捕获血液生态失衡的早期信号。利用这些早期信号开展微小残留病灶监测、免疫克隆追踪和循环细胞动态分析，能够对疾病状态实现实时、无创、动态地评估[16]。随着数字孪生模型应用前景初显，通过模拟个体血液生态演化轨迹，有望实现预测性诊断，而非依赖滞后性的结果判定，为血液疾病早诊早治提供科学路径。

在治疗方面，血液生态研究提出从单一靶点的清除到系统重建的范式升级。传统治疗多聚焦于抑制某一类细胞或阻断单一分子通路，易造成治疗后微环境重新塑造、恶性克隆逃逸或系统性免疫失衡等问题。血液生态强调恢复系统平衡的重要性，通过联合调控肿瘤细胞、免疫抑制细胞、血管与基质成分等微环境成员，同时影响代谢与应激网络，可在全局层面限制病理演化趋势。针对单倍体HSCT中抗胸腺细胞球蛋白最优剂量的探索显示，通过精准调控剂量可平衡免疫抑制与抗病毒免疫，由此证明恢复系统稳态对于抑制病理演化的核心价值[17]。此外，在HSCT领域，基于生态视角的细胞治疗理念正在形成，即不再只输注造血干细胞，而是移植物由造血干细胞、基质细胞、间充质干细胞及免疫调节细胞共同组成，借助多成员协同作用加速造血重建并降低aGVHD等风险。随着计算模型精度提升，未来可根据不同患者血液生态状态，设计动态可调治疗方案，实现个体化生态稳态恢复，而非追求完全清除某一异常克隆，从根本上提高治疗安全性与长期疗效。

由此，血液疾病诊疗将逐步摆脱经验式管理，迈向可预测、可组合、可重建的系统精准医疗新时代。血液生态提供了研究与实践的理论基础和系统框架，有望成为未来血液疾病研究、治疗设计及跨系统疾病干预的重要理论支柱。

六、血液生态与非血液系统疾病：跨系统机制与干预思路

血液生态研究不仅适用于血液系统疾病，也为理解非血液系统疾病提供了新的系统性框架。作为循环系统核心媒介，血液承载信号、细胞以及生物分子在全身范围内的跨器官流动，在疾病的发生、进展与转归中发挥关键作用。血液是获取疾病信息与风险评估的重要系统窗口。在实体瘤管理中，通过液体活检检测循环肿瘤DNA实现微小残留病灶的早期识别[16]，即便在影像学检查仍为阴性阶段，也可捕捉潜在复发或转移风险，体现血液对远端病变状态的实时反映能力。

越来越多证据提示血液生态失衡本身可驱动非血液系统疾病的病程演化。克隆性造血通过偏向性髓系输出诱导低度慢性炎症，可显著增加动脉粥样硬化与冠心病风险[18]，说明造血—免疫异常可跨越组织边界影响血管稳态。近期研究表明，骨髓造血偏向驱动外周免疫异常，并加重中枢神经系统炎症，促进多发性硬化（Multiple sclerosis，MS）病程进展，提出“造血—免疫—神经炎症轴”概念[19]，提示MS不只是局灶神经退行性疾病，而是血液生态失衡驱动的跨器官网络结果。

异体共生实验进一步证明了血液生态具有可转移性：将年轻与年老小鼠循环建立共享后，老年个体造血、肌肉和神经功能均出现改善，而年轻个体表现加速衰老[20]–[21]，说明衰老并非局限于细胞内源耗竭，而受到循环生态网络及其信号调控。衰老由此可被视为血液生态逐步失衡的系统性过程。心血管疾病研究进一步展现血液生态跨器官调控特性。心肌梗死后，局部组织损伤可通过体液因子远程诱导脾脏髓外造血增强，促进造血干/祖细胞扩增并偏向粒系分化，动员免疫细胞进入心脏参与组织修复[22]。PGI2-IP信号下降在其中发挥调节作用，造血细胞特异性敲除IP受体可改善修复结局，提示通过调整造血生态能够改善远端受损器官恢复，而不仅依赖局部治疗。

上述研究共同提示血液生态不仅反映病理状态，更能驱动跨器官病程演化，是探索全身性疾病机制的重要入口。未来疾病研究可围绕“器官损伤—造血—免疫循环”重建系统病理模型，通过扭转造血免疫失衡来改善远端器官修复，构建血液生态风险指标体系动态预测疾病进展，并利用人工智能解析血液生态指纹，实现不同个体风险、恢复能力与治疗响应的预测，为跨系统干预提供科学依据。同时，通过调节造血生态以重塑免疫微环境可增强抗肿瘤反应或改善组织保护，实现通过外周造血与循环调控影响心脏和脑组织修复的“远程治疗”策略。血液生态因此成为疾病研究范式创新的桥梁，推动精准医学从器官中心迈向系统中心，从局部治疗迈向生态重建，为非血液系统疾病提供新的诊断和治疗路径。

七、挑战与展望，迈向系统精准医学的生态医学框架

血液生态研究要真正支撑精准医学还面临多重挑战。其一，血液生态涉及多细胞类型、多时空状态与跨器官互作网络，不同研究平台、测序技术及注释标准差异显著，使数据在整合及跨队列比较中存在较高异质性，亟需建立统一的参考规范与质量控制体系。其二，多模态数据融合仍面临计算复杂性高、因果解析能力有限的问题，如何从转录组、蛋白组、代谢组、表观组及临床表型等多维数据中重建系统互作关系，需要依赖具备因果推断能力的新型计算模型。其三，血液生态具有高度动态性，而现有研究多基于静态采样，难以捕捉心梗后髓外造血启动等关键事件的时空演化轨迹，提示需要发展纵向与实时监测技术。其四，人工智能模型的可解释性与临床可落地性仍存在瓶颈，深度学习模型仍存在“黑箱”问题，临床安全应用需要建立可追溯、可解释的决策框架。

展望未来，血液生态作为系统医学与精准医学的基础框架，其学科站位与影响力正不断攀升。由于血液具有连接全身组织器官的天然纽带属性，其作为“系统镜像”解析复杂生理病理过程的独特价值正被广泛认可。这一科学属性将会吸引越来越多的临床医学、发育生物学及免疫学等多领域专家的深度参与，形成多学科共研的协作态势，从不同维度共同诠释血液生态在跨系统疾病中的核心逻辑。这种多学科范式的交叉融合，将驱动血液生态在复杂的系统性研究中得到更具广度与深度的解析。

与此同时，血液生态研究迈向系统科学阶段的核心驱动力在于高质量数据、先进计算模型与强大算力的协同作用。通过将海量的实验数据、临床表型与既有知识体系进行统一表征与推理，该领域正逐步迈向以数据与模型协同驱动的研究范式。在此基础上，结合多模态数据与人工智能推理能力，有望构建能够模拟血液生态长期演化过程的数字孪生系统，使假设检验、靶点筛选和治疗策略设计可在虚拟环境中预演，从而显著提升研究效率与转化潜力。在非血液系统疾病领域，围绕“器官损伤—造血—免疫调控”轴线等机制研究将持续深化，通过调控造血生态改善远端器官功能的干预策略有望逐步成熟，形成跨系统治疗新模式。这一方向的核心逻辑在于：血液系统不仅是受损器官的“镜像”映射，更是通过造血系统的重塑和免疫细胞的远程调遣，主动参与甚至主导了远端器官的病理演变。通过解析这一轴线上的信号传导与细胞命运决定机制，血液生态将从诊断窗口跨越为干预靶位。研究将聚焦于如何通过重塑造血微环境、纠正造血分化偏倚等手段，在源头上调节全身免疫稳态，从而实现对远端受损器官（如心血管、神经系统或实体肿瘤微环境）的功能修复与稳态重建。这种跨系统治疗新模式的成熟，将确立血液生态在多器官复杂疾病防治中的核心指导地位。在此基础上，通过对个体血液生态指纹的长期监测，临床上可实现对系统性疾病风险的极早期预警与分型，并据此制定涵盖细胞治疗与免疫干预的个体化精准方案，通过系统平衡的精准回溯与调控，最大限度地提升治疗效果并规避全身性风险。最后，随着多学科协作的深度融合，血液生态研究将会从描述性与关联性研究迈向可解释、可预测和可干预的系统科学新阶段。这一演进将为构建以系统稳态重建为核心的精准医学新范式奠定坚实的理论与实践基础。
